# Has alcohol consumption in England returned to pre‐COVID‐19 pandemic levels? A monthly population study, 2014 to 2024

**DOI:** 10.1111/add.70258

**Published:** 2025-11-23

**Authors:** Vera Helen Buss, Melissa Oldham, Sarah E. Jackson, Lion Shahab, Colin Angus, John Holmes, Jamie Brown

**Affiliations:** ^1^ Department of Behavioural Science and Health University College London London United Kingdom of Great Britain and Northern Ireland; ^2^ SPECTRUM Research Consortium Edinburgh United Kingdom of Great Britain and Northern Ireland; ^3^ School of Medicine and Population Health University of Sheffield Sheffield United Kingdom of Great Britain and Northern Ireland

**Keywords:** alcohol consumption, alcohol dependence, England, Great Britain, health inequities, socio‐economic factors

## Abstract

**Aim:**

To determine whether alcohol consumption in England had returned to pre‐pandemic levels by December 2024, after the initial rise in 2020 across the total population and subgroups.

**Design:**

Monthly representative surveys were conducted through face‐to‐face interviews until February 2020, and then by telephone.

**Setting:**

England, March 2014 to December 2024.

**Participants:**

208 010 adults aged 18+ living in private households.

**Measurements:**

Mean weekly alcohol consumption (in UK units), prevalence of risky drinking (Alcohol Use Disorders Identification Test for Consumption [AUDIT‐C] score≥5), and possible dependence (AUDIT‐C ≥ 11). Further measures included age, gender, and social grade.

**Findings:**

All outcomes increased in April 2020: prevalence of risky drinking by 30.3% (95% confidence interval [CI]: 26.8, 33.8, from 26.2% in February 2020 to 34.0% in April 2020), prevalence of possible dependence by 90.2% (95% CI: 62.2, 122.9, from 0.9% to 1.7%) and mean weekly alcohol consumption by 34.5% (95% CI: 31.0, 38.0, from 5.0 units to 6.6 units). When adjusting for the survey mode change from face‐to‐face to telephone interviews, the step changes between February and April 2020 remained but were substantially attenuated. The post‐pandemic trend declined more quickly than the pre‐pandemic trend for the prevalence of risky drinking (difference: −1.5%/year, 95% CI: −2.4, −0.6) and mean weekly alcohol consumption (difference: −2.4%/year, 95% CI: −3.3, −1.6), indicating a slow but incomplete return to pre‐pandemic levels. The trend in prevalence of possible dependence was similarly stable before and after the pandemic (difference: −1.3%/year, 95% CI: −6.2, 3.8). Alcohol consumption declined more slowly among people from less advantaged than from more advantaged social grades.

**Conclusions:**

The prevalence of risky drinking and mean weekly alcohol consumption in England appear to be trending towards pre‐pandemic levels but the prevalence of dependent drinking in England appears to have increased since the start of the pandemic and remains elevated compared with pre‐pandemic levels. Alcohol‐related inequalities may be worsening due to slower declines in consumption following the pandemic among less advantaged drinkers.

## INTRODUCTION

Alcohol consumption is a leading risk factor for preventable deaths worldwide [1]. In 2020, the United Kingdom (UK) saw a 19% rise in alcohol‐specific deaths (i.e. deaths from causes that are wholly attributable to alcohol), the largest increase since 2001 (start of the time series), from 11.8 alcohol‐specific deaths per 100 000 population in 2019 to 14.0 in 2020 [2]. Rates of alcohol‐specific deaths continued to rise thereafter, reaching record levels in 2023 [3]. Early in the pandemic, studies showed increases in the prevalence of risky drinking (operationalised as a score of 5 or above on the Alcohol Use Disorder Identification Test‐Consumption (AUDIT‐C) [4]) and mean alcohol consumption [5, 6, 7, 8, 9]. Some studies reported a polarisation in drinking, with some increasing and others decreasing their drinking [8, 9, 10], which was also observed in other countries [5, 11]. Generally, there is a lack of data published at any stage of the pandemic about trends in the prevalence of potential alcohol dependence (AUDIT‐C score ≥11).

Different sources, including the Health Survey for England [12], sales data from the British Beer and Pub Association [13] and UK alcohol duty data [14], indicate small population‐level changes are likely masking substantial subgroup‐level variations. Earlier studies, following the start of the coronavirus disease 2019 (COVID‐19) pandemic, showed that trends in the prevalence of risky drinking and mean weekly alcohol consumption were different across socio‐demographic groups [5, 6, 7, 8, 9, 15, 16]. One study found that increased alcohol consumption during the first lockdown in England was associated with being younger, female and having a higher income [8]. Another study reported that the increase in prevalence of risky drinking was more pronounced among women and people from less advantaged social grades [6]. The present study assessed, therefore, whether these subgroup differences have continued up to 2024. Understanding these variations can help to understand why alcohol‐specific deaths have continued to rise.

Further, the study examined potential differences between the three nations within Great Britain—England, Scotland and Wales. Generally, Scotland has the highest prevalence of risky drinking and the highest alcohol‐specific death rate in Great Britain [17, 18]. However, there are also policy differences between the nations which may have impacted consumption throughout and beyond the pandemic, such as minimum unit pricing, which has been in place in Scotland since 2018 and in Wales since March 2020 [19]. As the survey we used for analysis only collected data in Scotland and Wales since October 2020, the analyses comparing pre‐ and post‐pandemic trends focussed on England, while we looked at trends in all three nations since late 2020 as an additional analysis.

The specific research questions were:

1. Have the changes in: (1) prevalence of risky drinking (AUDIT‐C score ≥5); (2) prevalence of possible alcohol dependence (AUDIT‐C score ≥11); and (3) mean weekly alcohol consumption since the start of the pandemic returned to pre‐pandemic levels by December 2024, across the total adult population in England and within subgroups by age, gender and social grade?
2. How have trends in these outcomes changed among adults in the three nations of Great Britain from October 2020 to December 2024?

## METHODS

### Study design and participants

Data were from an ongoing cross‐sectional survey, the Alcohol Toolkit Study, with monthly data collection on drinking behaviours among adults in Great Britain [20, 21]. For the first research question, data were collected in England between May 2014 and December 2024 (the pre‐registered analysis plan stated January 2024, but the analyses were updated to include the most recent available data—results using data to January 2024 are available here and showed a similar pattern of results) [22]. For the second research question, data were collected in Great Britain between October 2020 (first data collection in Scotland and Wales) and December 2024 [21]. The sampling strategy is based on a combination of random location and quota sampling, which resulted in representative samples each month [23]. Because of the hybrid sampling approach, it is not possible to determine response rates. Each month, approximately 2400 households (in England ~1700) are randomly selected from 227 403 output areas across Great Britain (England ~170 000). Each area consists of approximately 300 households. The areas are stratified by an established geo‐demographic classification of the population called Acorn classification from Consolidated Analysis Centers (CACI). It classifies UK households, postcodes and neighbourhoods into six categories, 18 groups and 62 types using a combination of social factors and population behaviour [24]. A market research company conducts interviews with each one household member until quotas based on factors influencing the chances of being at home (e.g. age, gender and working status) are met and then provides the anonymised data to the research team [23].

Because of the first COVID‐19 lockdown in the United Kingdom, no data were collected in March 2020. After that, data collection switched from face‐to‐face to telephone interviews (addressed in unplanned sensitivity analyses described below) [25]. The sampling strategy is the same for face‐to‐face and telephone interviews. The Alcohol Toolkit Study comprises participants 16 years old and over, but during the pandemic, only those 18 years old and over were included. Therefore, this study set the age limit at 18, aligning with the UK's legal age of alcohol sales. The manuscript follows the Strengthening the Reporting of Observational Studies in Epidemiology (STROBE) statement [26]. The study protocol was pre‐registered [27].

### Outcome measures and covariates

All measures were self‐reported. The outcome measures were (1) the prevalence of drinking at risky levels; (2) the prevalence of drinking at possible dependence levels; and (3) mean weekly alcohol consumption (with 1 UK unit = 10 mL or 8 g of ethanol) across the total population. All of these were based on the AUDIT‐C questionnaire, which asks participants about their frequency of drinking, the quantity they consume on a typical occasion and their frequency of six or more units on a single occasion (see Data S1). Participants were categorised as drinking at risky levels if they scored five or above and as drinking at levels indicating possible alcohol dependence if they scored 11 or 12 using the AUDIT‐C score [4]. Mean weekly alcohol consumption was measured as a continuous variable using the AUDIT‐C questions 1 and 2 by multiplying drinking occasions per week by UK alcohol units consumed per typical drinking occasion. In an unplanned sensitivity analysis, we included an adjustment factor for AUDIT‐C question 3. The exact coding is provided in Data S1.

Time‐related variables included survey wave, two dummy variables representing pandemic effects, and month (treated as a categorical variable to account for seasonality). The survey wave was measured continuously, ranging from 1 (March 2014) to 130 (December 2024) for the first research question, and from 1 (October 2020) to 51 (December 2024) for the second research question. We omitted survey wave 73 (March 2020) when no data were collected. The first pandemic‐related variable was a step function to adjust for an abrupt increase in prevalence/consumption at the start of the pandemic. The World Health Organisation declared the COVID‐19 outbreak a global pandemic on 11 March and the UK Government announced the first lockdown on 23 March [28]. Therefore, the step function was coded 0 from May 2014 until February 2020, and 1 from April 2020 until December 2024. The second pandemic‐related variable was a ramp function to measure whether any change in the outcomes was on track to return to pre‐pandemic levels. It was coded 0 from May 2014 until February 2020 and then increased by 1 each month (from 1 in April 2020 to 46 in December 2024).

Socio‐demographic characteristics included age (18–29 years or 30+ years to differentiate emerging adulthood) [29], gender (women or men) and socio‐economic position (less or more advantaged). When reporting characteristics of study participants, we also provided the percentage who identified as non‐binary, but these were excluded when we stratified by gender because numbers were too small. Socio‐economic position was based on social grade using the National Readership Survey's occupation‐based classification [30]: more socio‐economically advantaged (ABC1: higher and intermediate managerial, administrative and professional, supervisory, clerical and junior managerial, administrative and professional) or less socio‐economically advantaged (C2DE: skilled manual workers, semi‐skilled and unskilled manual workers, state pensioners, casual and lowest grade workers, unemployed with state benefits only).

### Analysis

A complete case analysis was conducted. If the interviewer noted responses as ‘Do not know’ or ‘Refused’, these were classified as missing. The analysis was conducted on weighted data using raking [31] to match the population of England for the first research question, and England, Scotland and Wales, respectively, for the second research question (unweighted results in Data S1). Weights are computed each month by combining data from the UK Census, the Office for National Statistics mid‐year estimates and the annual National Readership Survey. The analysis was conducted in RStudio (version 2022.07.2, R version 4.2.1) using the *mgcv* package [32] for the modelling.

For the first research question, we used generalised additive models to measure the impact of the COVID pandemic in England on each outcome. For the first two outcomes, we used log‐binomial distributions in the models. Based on the histogram, the alcohol consumption data were zero‐inflated right‐skewed, therefore, we chose the Tweedie distribution for this model. Models included the survey wave, step function, ramp function and the seasonality variable that was modelled with a cyclic cubic spline function [33]. The models for prevalence of risky drinking and mean weekly alcohol consumption were repeated with stratification by age (18–29 years vs. 30+ years), gender (women vs. men) and social grade (ABC1/more advantaged vs. C2DE/less advantaged). We did not stratify the model for prevalence of drinking at possible dependence levels because numbers were small.

For the second research question, we estimated monthly time trends in England, Scotland and Wales from October 2020 to December 2024 for each of the three outcomes by using logistic regression (for binary outcomes) or generalised additive models with Tweedie distribution (for continuous outcome) with survey wave modelled using restricted cubic splines with three knots at the beginning, middle and end of the time series to allow for non‐linear trends [34]. Trends were then assessed descriptively by plotting the modelled estimates for each group over time. Specifications for all models are provided in the Data S1.

### Unplanned sensitivity analyses

After pre‐registering the protocol, we conducted a separate comparison of estimates derived from face‐to‐face and telephone interviews using data from two parallel survey waves in 2022 and 2024 [25]. Some estimates based on AUDIT‐C questions differed between survey modes. Notably, higher proportions of the face‐to‐face sample reported never drinking alcohol and never having six or more standard drinks on one occasion compared with the telephone sample [25]. As an unplanned sensitivity analysis, we aimed to assess potential impacts of the mode change on current study estimates. We re‐ran the models for the first research question on monthly aggregated data to which we added an adjustment factor for the survey mode change (more details in Data S1). We estimated 95% CI using a simulation‐based approach that accounted for the propagation of uncertainty of the adjustment factor. In each iteration (*n* = 1000), we sampled a value for the adjustment factor based on its estimated mean and standard error, which was then used to calculate the adjusted estimates. The CI bounds were then defined by the 2.5th and 97.5th percentile.

Second, we ran the analysis for research question 1 with mean weekly alcohol consumption adjusted for binge drinking (AUDIT‐C question 3) when assessing trends across the total population to see whether that changed the interpretation of the results (more details in Data S1). Third, we predicted when the pre‐ and post‐pandemic trends would intersect (i.e. when levels would return to pre‐pandemic levels), when assuming an ongoing linear trend. Fourth, we tested whether the interaction terms between socio‐demographic characteristics (age, gender and social grade) and step change, ramp effect and survey wave (first research question), as well as the interaction term between survey wave and nation (second research question), were statistically significant. In the latter case, we used the likelihood ratio and Wald tests to assess whether including the interaction term improved model fit. This cannot be directly derived from the model coefficients because we modelled time (survey wave) using restricted cubic splines.

## RESULTS

In total, 241 023 participants had complete data and were included in the analysis, which represents 98.0% of the total sample (*n* = 245 950, missing values per variable in Table S1). Participant characteristics for each nation are presented in Table 1 (unweighted in Table S2).

**TABLE 1:** Characteristics of participants (data weighted), for England: 2014–2024, for Scotland and Wales: 2020–2024.

	England (*N*_unweighted_ = 208 010)		Scotland (*N*_unweighted_ = 21 242)		Wales (*N*_unweighted_ = 11 771)	
	*n*	% (95% CI)	*n*	% (95% CI)	*n*	% (95% CI)
Women	101 697	50.8 (50.6–51.1)	10 890	51.7 (50.8–52.6)	6079	51.7 (50.4–52.9)
Men	105 860	48.8 (48.6–49.1)	10 074	47.8 (46.9–48.7)	5633	47.9 (46.5–49.2)
Non‐binary^a^	712	0.3 (0.3–0.4)	98	0.5 (0.4–0.6)	54	0.5 (0.3–0.6)
Age^b^ 18–29 y	41 971	20.2 (20.0–20.3)	3762	18.1 (17.2–19.1)	2052	17.4 (16.2–18.6)
Age^b^ 30+ y	166 298	79.8 (79.7–80.0)	17 310	82.1 (81.3–83.0)	9713	82.6 (81.4–83.8)
ABC1^c^	115 914	55.6 (55.4–55.9)	11 511	54.6 (53.7–55.5)	6356	54.0 (52.8–55.3)
AB	56 641	27.2 (27.0–27.4)	5257	24.9 (24.2–25.6)	2774	23.6 (22.6–24.5)
C1	59 273	28.5 (28.3–28.7)	6254	29.7 (28.9–30.4)	3582	30.4 (29.3, 31.5)
C2DE^d^	92 356	44.3 (44.1–44.6)	9561	45.4 (44.5–46.3)	5410	46.0 (44.7–47.2)
C2	43 653	21.0 (20.8–21.2)	4258	20.2 (19.5–20.9)	2297	19.5 (18.5–20.6)
D	30 276	14.5 (14.4–14.7)	3300	15.7 (14.8–16.5)	1803	15.3 (14.2–16.5)
E	18 427	8.8 (8.7–9.0)	2004	9.5 (9.0–10.0)	1309	11.1 (10.4–11.8)
Abstainer^e^	59 084	28.4 (28.2–28.6)	5021	23.8 (23.0–24.6)	2890	24.6 (23.5–25.6)

Abbreviations: ABC1, higher and intermediate managerial, administrative and professional, supervisory, clerical and junior managerial, administrative and professional; C2DE, skilled manual workers, semi‐skilled and unskilled manual workers, state pensioners, casual and lowest grade workers, unemployed with state benefits only.

^a^ Included as answer option since May 2017.

^b^ Median age, England: 47 years (interquartile range: 32–63), Scotland: 50 years (interquartile range: 33–64), Wales: 51 years (interquartile range: 34–65).

^c^ More advantaged social grades.

^d^ Less advantaged social grades.

^e^ Reporting no alcohol consumption.

### Trends and levels before and after the pandemic in England

**FIGURE 1:**
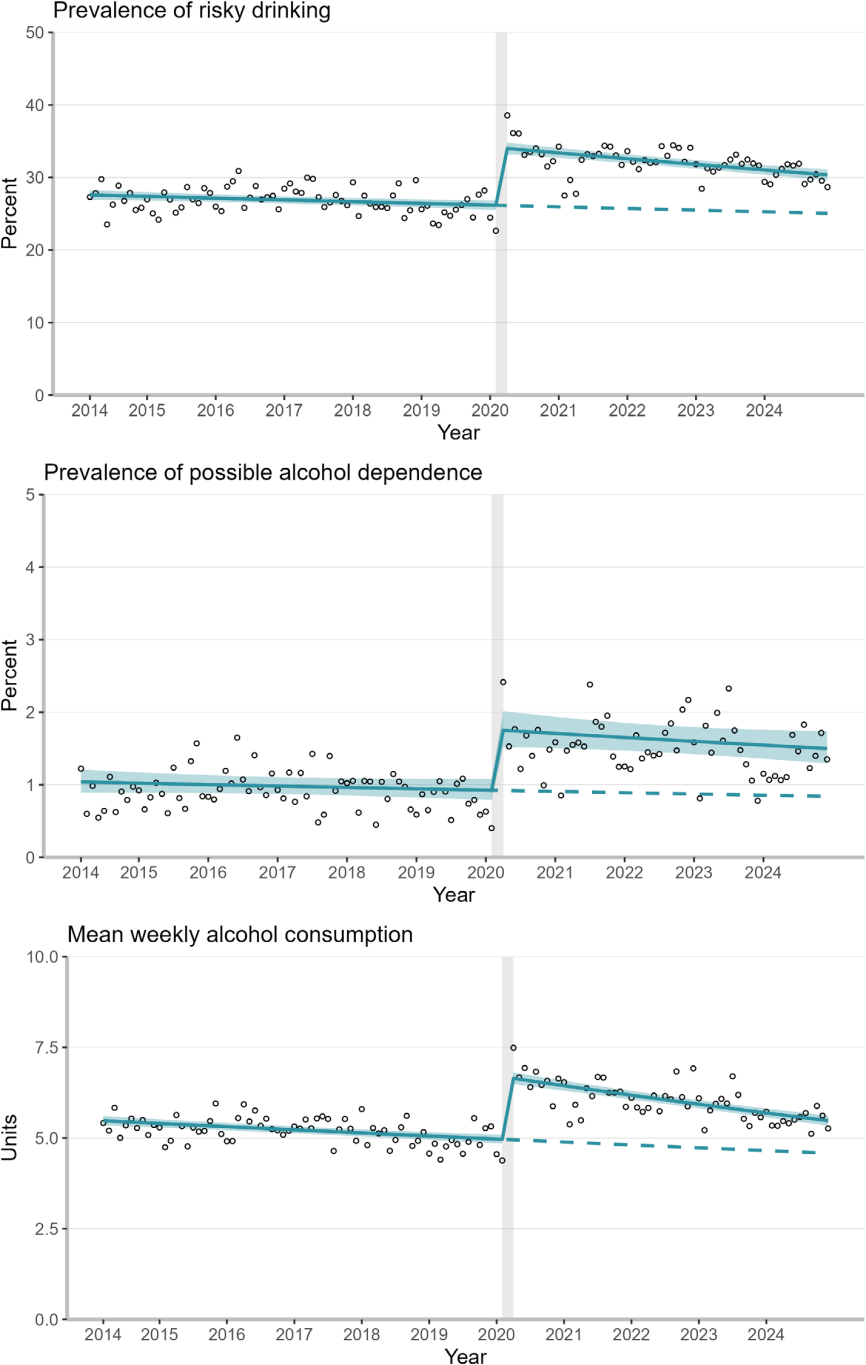
Trends in the weighted prevalence of risky drinking (top), weighted prevalence of possible alcohol dependence (middle) and weighted mean weekly alcohol consumption (bottom) among adults in England (*N*_unweighted_ = 208 010) between 2014 and 2024 with a step change after February 2020 (indicated with vertical grey line between February and April 2020). Modelled using generalised additive models. Shaded areas indicate 95% CIs. Dots show unmodelled values and dashed lines indicate continued pre‐trends. Parameter estimates are provided in Tables S3‐S4.

Before the start of the pandemic, the prevalence of risky drinking was declining by −0.9% (95% CI = −1.4 to −0.3) per year across all adults in England (Figure 1, Table 2, modelled estimates in Table S6). The pandemic onset was associated with a 30.3% (95% CI = 26.8–33.8) increase in prevalence, from 26.2% (95% CI = 25.5–26.8) to 34.0% (95% CI = 32.2–34.8). The post‐pandemic trend declined more quickly than the pre‐pandemic trend (difference: −1.5%/year, 95% CI = −2.4 to −0.6), indicating that prevalence of risky drinking has started to return to pre‐pandemic levels, with a predicted full return in October 2034 at a prevalence estimate of 22.8% (Table S8). When adjusting for the survey mode change from face‐to‐face to telephone interviews, the step change between February and April 2020 was 10.5% (95% CI = 3.3–16.4) (Tables S9–S11 and Figure S1). According to this estimate, pre‐pandemic levels would be achieved by February 2025 (Table S12).

**TABLE 2:** Percentage changes in weighted outcomes across all and subgroups in England (*N*_unweighted_ = 208 010).

	Pre vs. post, step change, % (95% CI)	Pre‐COVID trend, %/y (95% CI)	Pre‐ vs. post‐trend, Δ%/y (95% CI)
Prevalence of risky drinking			
All	30.3 (26.8–33.8)	−0.9 (−1.4 to −0.3)	−1.5 (−2.4 to −0.6)
Women	40.5 (34.0–47.4)	−0.9 (−1.9 to 0.0)	−2.3 (−3.8 to −0.8)
Men	25.0 (21.1–29.0)	−0.9 (−1.5 to −0.3)	−1.1 (−2.1 to −0.1)
Age 18–29 y	12.6 (6.6–19.0)	−0.8 (−1.8 to 0.3)	−0.8 (−2.6 to 1.0)
Age 30+ y	37.2 (33.0–41.5)	−0.9 (−1.6 to −0.3)	−1.7 (−2.7 to −0.7)
ABC1^a^	19.1 (15.5–23.0)	0.4 (−0.2 to 1.1)	−3.2 (−4.2 to −2.1)
C2DE^b^	52.4 (44.9–60.3)	−3.5 (−4.5 to −2.5)	1.6 (−0.1 to 3.2)
Prevalence of possible dependence			
All	90.2 (62.2–122.9)	−1.9 (−5.2 to 1.4)	−1.3 (−6.2 to 3.8)
Mean weekly alcohol consumption			
All	34.5 (31.0–38.0)	−1.6 (−2.1 to −1.1)	−2.4 (−3.3 to −1.6)
Women	41.3 (36.1–46.7)	−1.8 (−2.5 to −1.1)	−3.9 (−5.1 to −2.7)
Men	31.1 (26.5–35.8)	−1.6 (−2.2, −0.9)	−2.0 (−3.1, −0.8)
Age 18–29 y	25.6 (18.1–33.6)	−2.2 (−3.3 to −1.1)	−0.7 (−2.7 to 1.3)
Age 30+ y	35.6 (31.7–39.6)	−1.5 (−2.1 to −1.0)	−2.7 (−3.7 to −1.8)
ABC1^a^	25.3 (21.4–29.4)	−1.2 (−1.8 to −0.6)	−3.7 (−4.7 to −2.7)
C2DE^b^	51.2 (44.5–58.2)	−2.7 (−3.5 to −1.8)	−0.3 (−1.7 to 1.2)

*Note*: Absolute values for March 2014, February 2020, April 2020 and December 2024 can be found in Table S6.

Abbreviations: ABC1, higher and intermediate managerial, administrative and professional, supervisory, clerical and junior managerial, administrative and professional; C2DE, skilled manual workers, semi‐skilled and unskilled manual workers, state pensioners, casual and lowest grade workers, unemployed with state benefits only; COVID, coronavirus disease.

^a^ More advantaged social grades.

^b^ Less advantaged social grades.

The pre‐pandemic trend for prevalence of possible dependence was −1.9% per year (95% CI = −5.2 to 1.4). Between February and April 2020, the prevalence nearly doubled (90.2%, 95% CI = 62.2–122.9), from 0.9% (95% CI = 0.8–1.1) to 1.7% (95% CI = 1.5–2.0). There was no clear difference in the trends following the pandemic's start compared to before (−1.3%/year, 95% CI = −6.2 to 3.8), indicating that prevalence of possible dependence has not yet started to return to pre‐pandemic levels (predicted return in November 2043 at a prevalence estimate of 0.5%). In the survey mode adjusted analysis, the step change between February and April 2020 was 34.1% (95% CI = −24.2 to 65.9). When assuming this smaller step change, pre‐pandemic levels would be reached by December 2026.

There was a downward trend in mean weekly alcohol consumption (−1.6%/year, 95% CI −2.1 to −1.1) before the pandemic. Between February and April 2020, mean weekly consumption increased by 34.5% (95% CI = 31.0–38.0), from 5.0 units (95% CI = 4.8–5.1) to 6.6 (95% CI = 6.5–6.8). The difference in pre‐ and post‐trends was negative (−1.6%/year, 95% CI = −2.1 to −1.1), suggesting that mean weekly alcohol consumption has started to return to pre‐pandemic levels, with a predicted full return in June 2030 at 4.1 units per week. After adjusting for the survey mode effect, the step change between February and April 2020 was 19.6% (95% CI = 10.6–24.5). Subsequently, the pre‐pandemic levels would be reached by September 2025.

**FIGURE 2:**
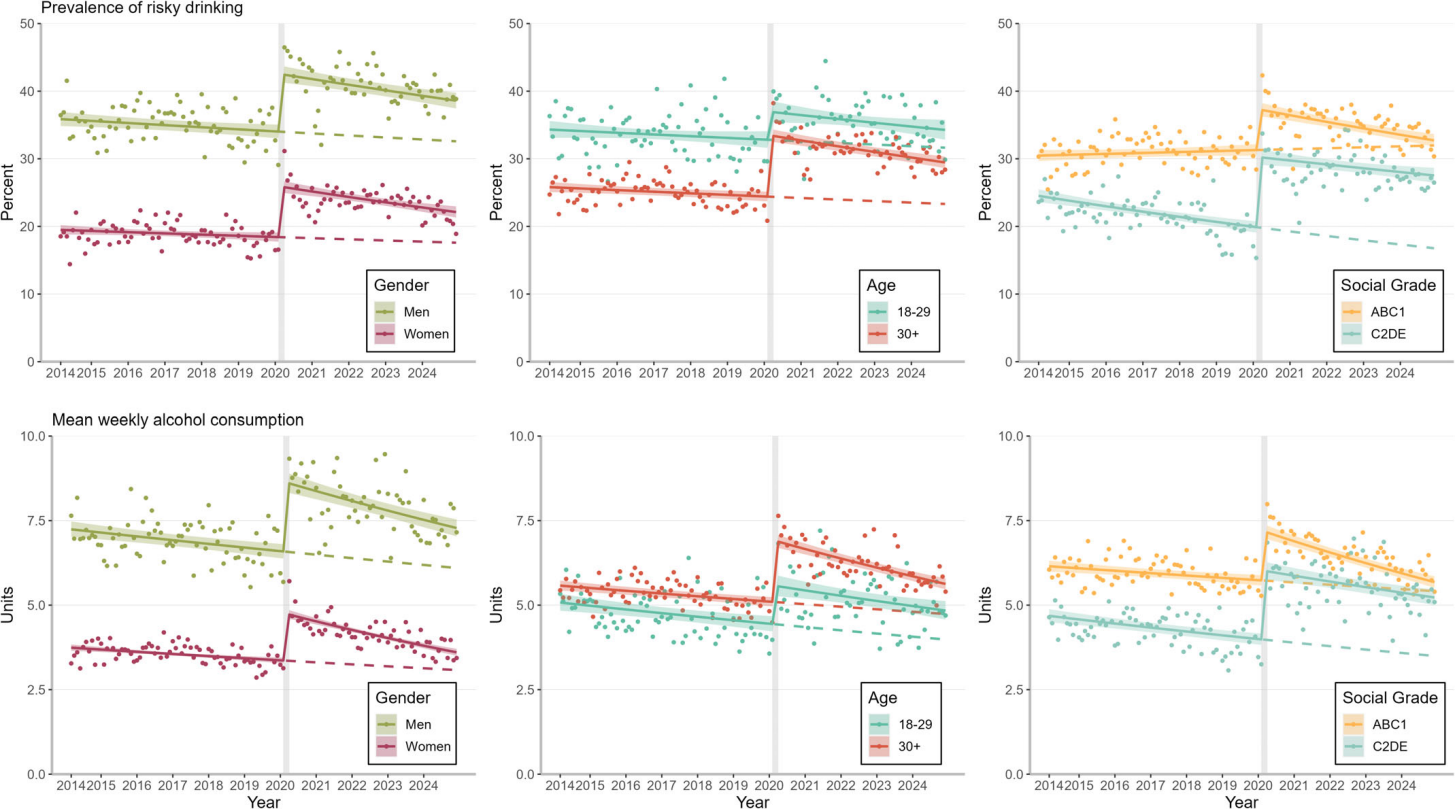
Trends in the weighted prevalence of risky drinking (left) and weighted mean weekly alcohol consumption (right) among adults in England (*N*_unweighted_ = 208 010) between 2014 and 2024 with a step change in February 2020 (indicated with vertical grey line between February and April 2020)—stratified by gender (left), age (middle) and social grade (right). Modelled using generalised additive models. Shaded areas indicate 95% CIs. Dots show unmodelled values and dashed lines indicate continued pre‐trends. ABC1 indicates more advantaged social grades and C2DE less advantaged social grades. Parameter estimates are provided in Tables S3‐S4.

There were differences in the return to pre‐pandemic levels for prevalence of risky drinking and mean weekly alcohol consumption by gender, age and social grade (see Table S5 for interaction terms). Women generally had a lower prevalence of risky drinking and consumed fewer units of alcohol per week than men (Figure 2). However, the relative increases between February and April 2020 were more pronounced among women than men for both outcomes (Table 2). The differences in pre‐ and post‐trends were negative for both outcomes and among both subgroups, which indicated a return to pre‐pandemic levels. As post‐pandemic trends showed a steeper decline for women, pre‐and post‐trends were predicted to converge earlier for women (in June 2032 for risky drinking and December 2027 for consumption) than for men (in September 2036 and October 2031). When adjusting for the change of survey mode, the step increase between February and April 2020 was still more pronounced among women than men, with returns to pre‐pandemic levels predicted between November 2024 (for consumption among women) and September 2027 (for consumption among men) (Tables S12–S13 and Figure S2).

Those aged ‐to‐29‐years had a higher overall prevalence of risky drinking than those 30 years and over, but the latter group had a higher weekly alcohol consumption. The relative increase in both outcomes was larger in the older than the younger age group. The differences in pre‐ and post‐trends were both negative for the older age group, suggesting that the measures have started to return to pre‐pandemic levels (predicted return in March 2035 for risky drinking and October 2029 for consumption). For the younger age group, the pre‐ and post‐trends were comparable, implying a prolonged return to pre‐pandemic levels despite the smaller increase at the start of the pandemic (predicted return in July 2033 for risky drinking and November 2041 for consumption). When adjusting for the change in survey mode, in the younger age group, there was no significant change in outcomes between February and April 2020, but in the older age group, there was still a significant increase in both outcomes, with a return to pre‐pandemic levels predicted for February 2025 for risky drinking and May 2026 for consumption.

People from more advantaged social grades had a higher prevalence of risky drinking and consumed more units of alcohol per week than people from less advantaged social grades, but between February and April 2020, the relative increases in both were much larger among those in less advantaged social grades than those from more advantaged social grades. The differences in pre‐ and post‐trends were both negative among those in more advantaged social grades, indicating that drinking behaviours started to return to pre‐pandemic levels (predicted return in October 2025 for risky drinking and February 2026 for consumption). In contrast, among those in less advantaged social grades, the difference in trends for prevalence of risky drinking was positive, albeit with slight uncertainty (1.6%/year, 95% CI = −0.1 to 3.2) and, for mean weekly alcohol consumption, pre‐ and post‐trends were comparable (−0.3%/year, 95% CI = −1.7 to 1.2), indicating that the measures were not on track to return to pre‐pandemic levels (predicted return in April 2056 for consumption and no return expected for risky drinking based on post‐pandemic linear trend).

When adjusting for the change in survey mode, there was still a significant increase in outcomes among more advantaged social grades between February and April 2020 (predicted return in February 2025 for risky drinking and April 2026 for consumption). In contrast, among less advantaged social grades, there was no longer a significant change in prevalence of risky drinking or mean weekly consumption. However, the post‐pandemic trend for the prevalence of risky drinking appeared to decline at a slower rate than the pre‐trend. Therefore, after March 2025 (i.e. when the two trends overlap), the levels would be higher than would have been expected if the pre‐pandemic trend had continued.

### Trends in England, Scotland and Wales since October 2020

**FIGURE 3:**
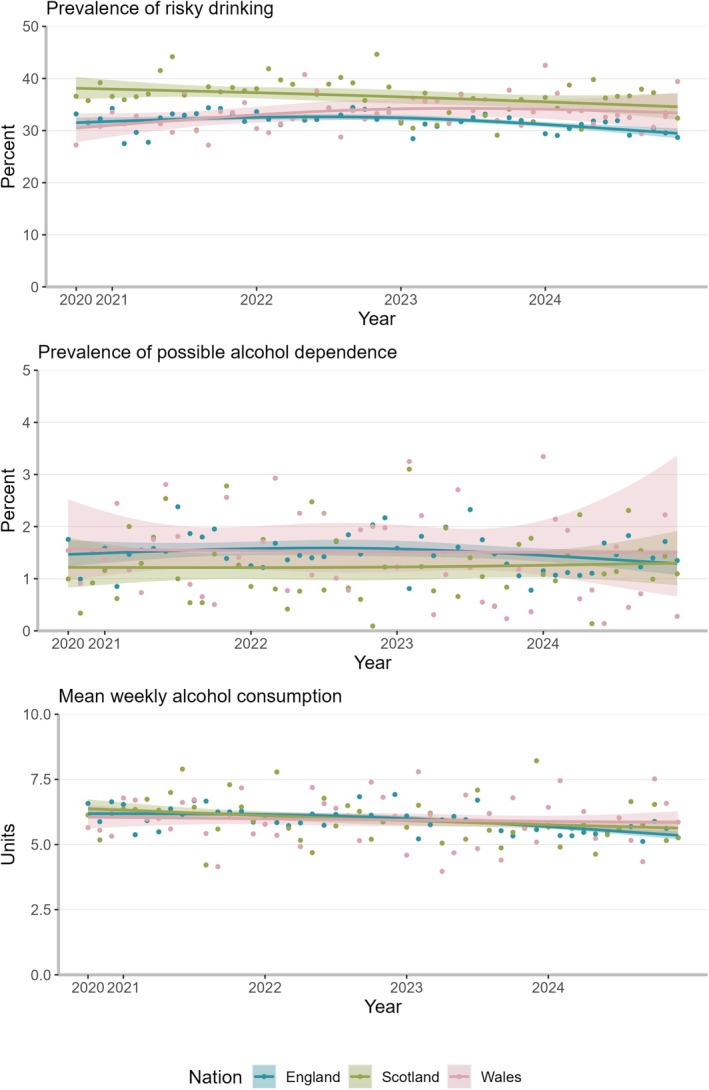
Trends in the weighted prevalence of risky drinking (top), weighted prevalence of possible alcohol dependence (middle) and weighted mean weekly alcohol consumption (bottom) among adults England (*N*_unweighted_ = 79 444), Scotland (*N*_unweighted_ = 21 242) and Wales (*N*_unweighted_ = 11 771) between 2020 and 2024. Modelled using restricted cubic splines with three knots. Shaded areas indicate 95% CIs. Dots show unmodelled values [prevalence of possible alcohol dependence estimate (6.3%) for Wales in October 2024 not shown].

Since October 2020, there were significant differences in the trends between the three nations, as demonstrated by the likelihood ratio test (2logLR = 104.7, *P* < 0.001) and the Wald test (F = 20.7 on 6 and 122 448 d.f., *P* < 0.001), which show that including an interaction term between time and nation improved model fit. The estimated absolute prevalence of risky drinking in October 2020 was higher in Scotland (38.1%, 95% CI = 36.0–40.3) than in England (31.5%, 95% CI = 30.6–32.5) and Wales (30.5%, 95% CI = 27.8–33.3) (Figure 3, modelled estimates in Table S7). Over time there was a potential increase in Wales and a potential decrease in Scotland, while England might have first seen a slight increase and then a decrease. By December 2024, the prevalence estimates were similar in Scotland (34.6%, 95% CI = 32.1–37.1) compared with Wales (33.4%, 95% CI = 29.8–37.2) and lower in England (29.5%, 95% CI = 28.5–30.4). The prevalence of potential alcohol dependence was overall relatively stable throughout the time series in all three nations. Mean weekly alcohol consumption decreased in England [from 6.2 units (95% CI = 6.0–6.4) to 5.4 units (95% CI = 5.2–5.5)] and Scotland [from 6.4 units (95% CI = 6.0–6.7) to 5.6 units (95% CI = 5.3–5.9)], but not in Wales [6.1 units (95% CI = 5.6–6.5) and 5.8 units (95% CI = 5.4–6.3)].

### Sensitivity analyses

The results from the unweighted sensitivity analyses and from the analysis adjusting mean weekly alcohol consumption for binge drinking were consistent with the main results (Figures S3–S6 and Tables S15–16).

## DISCUSSION

### Summary

For risky drinking and mean weekly alcohol consumption, the differences in pre‐ and post‐trends were negative, suggesting that these measures may return to pre‐pandemic levels. For prevalence of possible dependence, there was no clear difference in the trends before and after the pandemic's start, suggesting that it has not yet started to return to pre‐pandemic levels. However, there is uncertainty about the extent to which levels increased at the start of the pandemic, because the observed increases might be substantially attributable to the survey mode change.

The most noteworthy subgroup differences were by social grade. Although more advantaged groups consistently showed a higher prevalence of risky drinking and mean weekly alcohol consumption, the increase in less advantaged groups was much greater since the start of the pandemic, narrowing the difference between the groups. However, when adjusting for the change in survey mode, the extent of the change in prevalence of risky drinking or mean weekly alcohol consumption among less advantaged groups at the start of the pandemic was uncertain. Potentially, the observed difference could be explained by less advantaged people reporting higher alcohol consumption in telephone than in face‐to‐face interviews. Nevertheless, since then, prevalence of risky drinking appears to have declined at a slower pace in less advantaged groups compared to before the pandemic, suggesting that, at a minimum, more people are drinking at risky levels than would have been expected based on pre‐pandemic trends.

In October 2020, when data collection started across Great Britain, Scotland had a higher prevalence of risky drinking than England and Wales. However, it decreased in Scotland and potentially in England, while there was a potential slight increase in Wales. The prevalence estimates for possible alcohol dependence were similar across the nations. In England and Scotland, mean weekly alcohol consumption slightly decreased over time, but not in Wales.

### Strengths and limitations

The study's strengths include the large, representative sample and continuous monthly data collection, spanning 10 years, including 4 years after the onset of the pandemic. An unavoidable limitation of the study is that the data collection mode changed when the pandemic started. People seem to report lower alcohol consumption in face‐to‐face compared to telephone interviews, with the exact magnitude of the mode effect being uncertain [25]. We conducted an unplanned sensitivity analysis adjusting for this effect. It provides useful insights into potential mode effects on the outcomes, although it cannot fully offset the true effect. Accounting for the adjustment reduced the relative increase in this study's outcomes measured at the start of the pandemic, indicating that part of the increase between February and April 2020 may be because of the survey mode change. It also showed that the change in survey mode had different effects on the individual subgroups. For example, although more socio‐economically advantaged groups appeared to report similar alcohol consumption via face‐to‐face and telephone interviews, less socio‐economically advantaged groups reported substantially lower consumption in face‐to‐face interviews. Stigma and privacy concerns may be reduced by shifting away from face‐to‐face interviewing, with these effects potentially being more pronounced among less advantaged groups and for sensitive outcomes.

However, rising alcohol‐specific mortality rates since the pandemic started [3, 35] provide face validity for increases in consumption and risky drinking, suggesting that some of the increases measured in this study are genuine rather than just artefacts of the mode change. Changes in drinking behaviour were also observed in other countries, including consolidation or intensification of high‐level drinking patterns among people with pre‐pandemic high drinking levels and increased mortality rates [36, 37, 38, 39].

Further limitations include smaller sample sizes in Scotland and Wales, which reduce the ability to detect differences between nations, and likely under‐representation of people experiencing alcohol dependence and those from the least advantaged groups, which is a common issue in surveys [40, 41].

### Implications

If the rise in alcohol consumption observed at the start of the pandemic has not returned to pre‐pandemic levels, we would expect that alcohol‐related ill health and mortality rates will continue to be higher than before the pandemic [7]. The increase in alcohol‐specific mortality is likely because of a combination of continued increase in drinking among high‐risk groups (i.e. less advantaged groups, people who already had alcohol use disorder or potentially relapse for those who were previously abstinent) and a lack of access to prevention and treatment services during the pandemic [42, 43, 44, 45].

A further important consideration is that the acute phase of the pandemic was followed by a cost‐of‐living crisis in the UK, with inflation rising sharply between March 2021 and October 2022. Research has shown that economic crises also impact alcohol consumption, for example, while less disposable income can lead to reduced consumption, increased psychological distress among more vulnerable populations because of a crisis can increase harmful drinking [46]. Despite a decline in overall alcohol sales in the UK since the start of the cost‐of‐living crisis [47], it may contribute to sustaining the rise in alcohol‐related harm, again, particularly among less advantaged groups who experienced greater increases in psychological distress [48].

Therefore, new alcohol control policies with a focus on narrowing inequalities are required to reduce alcohol‐related harm in Great Britain, such as those to reduce the affordability, accessibility and advertising of alcohol [49, 50]. In relation to affordability, the implementation of minimum unit pricing is currently being discussed in England [49, 51] and was increased in Scotland from 50 pence to 65 pence per unit in October 2024 [52]. Regarding advertising, the Scottish Government commissioned an evidence review on potential alcohol advertising and marketing restrictions (e.g. ban on alcohol sports sponsorship) in September 2024 [52].

The sharp rise in people drinking at potential dependence levels is concerning and suggests that, among those who were already at considerable risk of alcohol‐related harm, some further increased their risk. This is in line with another study showing alcohol consumption increased in those drinking more heavily, but fell in those drinking moderately in England between 2020 and 2021 [7]. These findings suggest that more funding for treatment services may be required.

## CONCLUSION

The prevalence of risky drinking, possible dependence and mean weekly alcohol consumption all increased substantially between February and April 2020 after the onset of the COVID‐19 pandemic. The prevalence of risky drinking and mean weekly alcohol consumption appear to be trending toward pre‐pandemic levels, with returns to pre‐pandemic projections possibly in 2034 and 2030, respectively (or in each case in 2025 after adjustment for a change in the survey mode). In contrast, the prevalence of possible dependence appears to remain at increased levels, which is consistent with increases in alcohol‐related mortality observed in England. However, there is uncertainty because sensitivity analyses suggested the immediat e pandemic increases might be in part attributable to a survey mode change from face‐to‐face to telephone.

## AUTHOR CONTRIBUTIONS

**Vera Helen Buss:** Conceptualization (lead); data curation (lead); methodology (lead); visualization (lead); writing—original draft (lead). **Melissa Oldham:** Conceptualization (supporting); methodology (supporting); writing—review and editing (equal). **Sarah E. Jackson:** Conceptualization (supporting); methodology (supporting); writing—review and editing (equal). **Lion Shahab:** Conceptualization (supporting); funding acquisition (equal); methodology (supporting); writing—review and editing (equal). **Colin Angus:** Conceptualization (supporting); methodology (supporting); writing—review and editing (equal). **John Holmes:** Conceptualization (supporting); methodology (supporting); writing—review and editing (equal). **Jamie Brown:** Conceptualization (supporting); data curation (supporting); funding acquisition (equal); methodology (supporting); validation (lead); writing—review and editing (supporting).

## DECLARATION OF INTERESTS

J.B. has received unrestricted research funding from Pfizer and J&J, who manufacture smoking cessation medications. L.S. has received honoraria for talks, unrestricted research grants and travel expenses to attend meetings and workshops from manufactures of smoking cessation medications (Pfizer; J&J) and has acted as paid reviewer for grant awarding bodies and as a paid consultant for health care companies. All authors declare no financial links with the alcohol industry or their representatives.

## ETHICS STATEMENT

The University College London Ethics Committee granted ethical approval for the Alcohol Toolkit Study (ID 0498/001).

## Supporting information


**Data S1.** Supplementary Information.

## Data Availability

De‐identified participant data and the command syntax for the statistical analyses will be available with publication on the Open Science Framework: https://osf.io/6hvkf/.
