# Supplementary material for: Has alcohol consumption in England returned to pre‐COVID‐19 pandemic levels? A monthly population study, 2014 to 2024
Source: Addiction. 2025 Nov 23;121(4):839–50. doi: 10.1111/add.70258 (PMC12980288; doi:10.1111/add.70258)
Supplement: Supplementary file 1 — Data S1. Supplementary Information. [file ADD-121-839-s001.pdf]

Supplementary material

Article: Has alcohol consumption in England returned to pre-COVID-19 pandemic levels? A monthly population study, 2014-2024

Table of Contents

1. Outcome measures 1
2. Missing values 2
3. Unweighted characteristics of participants 2
4. Model specifications 3
5. Modelled weighted estimates 4
6. Analysis adjusted for survey mode change 5
7. Unweighted trends 10
8. Mean weekly alcohol consumption adjusted for binge drinking 14

1. Outcome measures

Alcohol Use Disorder Identification Test – Consumption (AUDIT-C)

The survey introduced these questions with the following:

"These first few questions ask about the alcohol you have drunk in the last 6 months, including about how many standard drinks you have consumed. Please note that 1 standard drink equals 1 unit of alcohol. So, for example, a small glass of wine or a single measure of spirits is 1 standard drink, while a pint of regular beer or lager is equal to 2 standard drinks or 2 units, and a bottle of wine is equal to 9 units. If you are unsure, please ask me to help you work it out.

Please be aware that all your answers will be handled confidentially."

AUDIT-C question 1: *"How often do you have a drink containing alcohol?*

- i. *Never [value: 0]*
- ii. *Monthly or less [value: 1]*
- iii. *2 to 4 times a month [value: 2]*
- iv. *2 to 3 times a week [value: 3]*
- v. *4 to 5 times a week [value: 4]*
- vi. *6 or more times a week [value: 4]*
- vii. *Don't know*
- viii. *Refused"*

The following questions are asked to all except those who answered (i), (vii), or (viii) to AUDIT-C question 1.

AUDIT-C question 2: *"How many standard drinks containing alcohol do you have on a typical day when you are drinking?*

- i. *1 to 2 [value: 0]*
- ii. *3 to 4 [value: 1]*
- iii. *5 to 6 [value: 2]*
- iv. *7 to 9 [value: 3]*
- v. *10 to 12 [value: 4]*
- vi. *13 to 15 [value: 4]*
- vii. *16 or more [value: 4]*
- viii. *Don't know*
- ix. *Refused"*

AUDIT-C question 3: *"How often do you have six or more standard drinks on one occasion?*

- i. *Never [value: 0]*
- ii. *Less than monthly [value: 1]*
- iii. *Monthly [value: 2]*
- iv. *Weekly [value: 3]*
- v. *Daily or almost daily [value: 4]*
- vi. *Don't know*
- vii. *Refused"*

Risky drinking – binary variable, measured using the AUDIT-C (i.e., AUDIT questions 1-3), with a score of 5 or above indicating risky drinking.

Possible alcohol dependence – binary variable, measured using the AUDIT-C, with a score of 11 or 12 indicating possible alcohol dependence.

Mean weekly alcohol consumption – continuous variable, based on AUDIT questions 1 and 2 (see above).

Drinking occasions per week will be coded as:

- Never: 0,
- monthly or less: 0.25,
- 2–4 times per month: 0.75,
- 2–3 times per week: 2.5,
- 4–5 times per week: 4.5,
- 6+ times per week: 6.5.

Units consumed per drinking occasion will be coded as:

- 1–2: 1.5,
- 3–4: 3.5,
- 5–6: 5.5,
- 7–9: 8,
- 10–12: 11,
- 13–15: 14,
- 16+: 21.

Weekly units of alcohol consumed will be calculated as drinking occasions per week times units consumed per drinking occasion.

2. Missing values

Table S1: Missing values for each variable included in the analysis.

	Missing values, n (%)		
	England (N=211,769)	Scotland (N=22,035)	Wales (N=12,146)
AUDIT-C[1]	3500 (1.7)	739 (3.4)	350 (2.9)
Weekly alcohol consumption	3016 (1.4)	619 (2.8)	278 (2.3)
Age	0 (0.0)	0 (0.0)	0 (0.0)
Gender	276 (0.1)	57 (0.3)	27 (0.2)
Social grade	0 (0.0)	0 (0.0)	0 (0.0)

[1] Alcohol use disorder identification test – consumption.

3. Unweighted characteristics of participants

Table S2: Characteristics of participants (data unweighted)

	**England** (N=208,010)		**Scotland** (N=21,242)		**Wales** (N=11,771)	
	n	%	n	%	n	%
Women	103736	49.9	11067	52.1	5999	51.0
Men	103560	49.8	10077	47.4	5718	48.6
Non-binary	714	0.3	98	0.5	54	0.5
Age[1] 18-29	40243	19.3	1807	10.2	819	8.6
Age[1] 30+	167767	80.7	14643	89.8	8239	91.4
ABC1[2]	127787	61.4	10736	66.0	5728	63.8
- AB	51868	24.9	5803	27.3	3135	26.6
- C1	75919	36.5	8221	38.7	4373	37.2
C2DE[3]	80223	38.6	5714	34.0	3330	36.2
- C2	37068	17.8	3188	15.0	1809	15.4
- D	23113	11.1	1639	7.7	889	7.5
- E	20042	9.6	2391	11.3	1571	13.3
Abstainer[4]	60075	28.9	2157	22.8	3668	24.4

[1] Included as answer option since May 2017; [2] median age, England: 50 years (interquartile range: 33-65), Scotland: 57 years (interquartile range: 42-69), Wales: 61 years (interquartile range: 46-73); [3] more advantaged social grades; [3] less advantaged social grades; [4] reporting no alcohol consumption.

4. Model specifications

Risky drinking

- Family: quasibinomial
- Link function: log
- Smooth term: cyclic cubic regression spline
- Method: restricted maximum likelihood
- Optimizer: outer newton
- Formula: risky drinking ~ step + ramp + survey wave + s(seasonality, k = 12)

Table S3: Parameter estimates for risky drinking model.

	Step, estimate (SE[1]), p-value	Ramp estimate (SE), p-value	Survey wave estimate (SE), p-value	s(seasonality) edf[2], p-value
All	0.264 (0.014), p<0.001	-0.001 (0.000), p=0.001	-0.001 (0.000), p=0.001	5.44, p<0.001
Women	0.340 (0.024), p<0.001	-0.002 (0.001), p=0.003	-0.001 (0.000), p=0.057	1.24, p=0.094
Men	0.223 (0.016), p<0.001	-0.001 (0.000), p=0.031	-0.001 (0.000), p=0.006	6.55, p<0.001
Age 18-29	0.119 (0.028), p<0.001	-0.001 (0.001), p=0.377	-0.001 (0.000), p=0.150	0.55, p=0.187
Age 30+	0.316 (0.016), p<0.001	-0.001 (0.000), p=0.004	-0.001 (0.000), p=0.004	6.42, p<0.001
ABC1[3]	0.175 (0.016), p<0.001	-0.003 (0.000), p<0.001	0.000 (0.000), p=0.167	4.20, p=0.003
C2DE[4]	0.422 (0.026), p<0.001	0.001 (0.001), p=0.059	-0.003 (0.000), p<0.001	2.33, p=0.002

[1] Standard error; [2] estimated degrees of freedom; [3] more advantaged social grades; [4] less advantaged social grades.

Possible alcohol dependence

- Family: quasibinomial
- Link function: log
- Method: restricted maximum likelihood
- Optimizer: outer newton
- Smooth term: cyclic cubic regression spline
- Formula: possible dependence ~ step + ramp + survey wave + s(seasonality, k = 12)
- Step estimate: 0.643 (standard error: 0.081), p<0.001
- Ramp estimate: -0.001 (standard error: 0.002), p=0.605
- Survey wave estimate: -0.002 (standard error: 0.001), p=0.255
- S(seasonality) estimated degrees of freedom: 8.99, p=0.014

Mean weekly alcohol consumption

- Family: Tweedie(p=1.635)
- Link function: log
- Smooth term: cyclic cubic regression spline
- Method: restricted maximum likelihood
- Optimizer: outer newton
- Formula: mean weekly alcohol consumption ~ step + ramp + survey wave + s(seasonality, k = 12)

Table S4: Parameter estimates for mean weekly alcohol consumption model.

	Step estimate (SE[1]), p-value	Ramp estimate (SE), p-value	Survey wave estimate (SE), p-value	s(seasonality) edf[2], p-value
All	0.296 (0.013), p<0.001	-0.002 (0.000), p=0.001	-0.001 (0.000), p<0.001	6.964, p<0.001
Women	0.340 (0.024), p<0.001	-0.003 (0.001), p<0.001	-0.002 (0.000), p<0.001	0.18, p=0.396
Men	0.271 (0.018), p<0.001	-0.002 (0.000), p=0.001	-0.001 (0.000), p<0.001	7.30, p<0.001
Age 18-29	0.228 (0.032), p<0.001	-0.001 (0.001), p=0.498	-0.001 (0.001), p<0.001	4.36, p<0.001
Age 30+	0.304 (0.015), p<0.001	-0.002 (0.000), p<0.001	-0.002 (0.000), p<0.001	7.62, p<0.001
ABC1[3]	0.226 (0.016), p<0.001	-0.003 (0.000), p<0.001	-0.001 (0.000), p<0.001	5.02, p<0.001
C2DE[4]	0.413 (0.023), p<0.001	0.000 (0.001), p=0.740	-0.002 (0.000), p<0.001	7.79, p<0.001

[1] Standard error; [2] estimated degrees of freedom; [3] more advantaged social grades; [4] less advantaged social grades.

Table S5: Interactions between sociodemographic characteristics and step, ramp, and survey wave.

	**Risky drinking, p-value for interaction**			**Alcohol consumption, p-value for interaction**		
	Step	Ramp	Survey wave	Step	Ramp	Survey wave
Gender	<0.001	0.206	0.856	0.009	0.020	0.798
Age	<0.001	0.388	0.694	0.017	0.067	0.328
Social grade	<0.001	<0.001	<0.001	<0.001	<0.001	0.002

5. Modelled weighted estimates

Table S6: Modelled weighted estimates for prevalence of risky drinking, prevalence of possible alcohol dependence, and mean weekly alcohol consumption among all adults ($N_{unweighted}$=208,010) and subgroups in England in March 2014, February 2020, April 2020, and December 2024. Time modelled linearly.

	Sample	March 2014	February 2020	April 2020	December 2024
Prevalence of risky drinking, % (95% CI)	All	27.6 (26.9, 28.3)	26.2 (25.5, 26.8)	34.0 (33.2, 34.8)	30.4 (29.6, 31.1)
	Women	19.5 (18.8, 20.2)	18.4 (17.8, 19.1)	25.8 (24.9, 26.7)	22.8 (22.2, 23.5)
	Men	35.9 (34.9, 36.9)	34.0 (33.0, 35.1)	42.4 (41.2, 43.7)	38.6 (37.4, 39.8)
	Age 18-29	34.3 (33.1, 35.6)	32.8 (31.6, 34.1)	36.9 (35.4, 38.5)	34.3 (32.8, 35.8)
	Age 30+	25.8 (25.1, 26.6)	24.4 (23.7, 25.1)	33.4 (32.5, 34.3)	30.2 (29.4, 31.0)
	ABC1[1]	30.5 (29.7, 31.3)	31.3 (30.4, 32.2)	37.2 (36.2, 38.2)	32.7 (31.8, 33.7)
	C2DE[2]	24.5 (23.6, 25.5)	19.9 (19.1, 20.7)	30.2 (29.1, 31.4)	28.0 (27.2, 29.0)
Prevalence of possible dependence, % (95% CI)	All	1.0 (0.9, 1.2)	0.9 (0.8, 1.1)	1.7 (1.5, 2.0)	1.5 (1.3, 1.7)
Mean weekly alcohol consumption, units (95% CI)	All	5.5 (5.3, 5.6)	5.0 (4.8, 5.1)	6.6 (6.5, 6.8)	5.5 (5.3, 5.6)
	Women	3.7 (3.7, 3.8)	3.4 (3.3, 3.4)	4.7 (4.7, 4.9)	3.8 (3.7, 3.9)
	Men	7.2 (7.0, 7.5)	6.6 (6.4, 6.8)	8.6 (8.3, 8.9)	7.3 (7.0, 7.5)
	Age 18-29	5.1 (4.8, 5.3)	4.4 (4.2, 4.7)	5.6 (5.3, 5.9)	4.8 (4.6, 5.1)
	Age 30+	5.6 (5.4, 5.7)	5.1 (5.0, 5.2)	6.9 (6.7, 7.1)	5.9 (5.7, 6.0)
	ABC1[1]	6.1 (6.0, 6.3)	5.7 (5.6, 5.9)	7.1 (6.9, 7.4)	5.7 (5.5, 5.9)
	C2DE[2]	4.7 (4.5. 4.9)	4.0 (3.8, 4.2)	6.0 (5.7, 6.3)	5.4 (5.2, 5.6)

[1] More advantaged social grades; [2] less advantaged social grades.

Table S7: Modelled weighted estimates for prevalence of risky drinking, prevalence of possible alcohol dependence, and mean weekly alcohol consumption among adults in England ($N_{unweighted}$=79,444), Scotland ($N_{unweighted}$=22,035), and Wales ($N_{unweighted}$=11,771) in October 2020 and December 2024. Time modelled using restricted cubic splines.

	Nation	October 2020	December 2024
Prevalence of risky drinking, % (95% CI)	England	31.5 (30.6, 32.5)	29.5 (28.5, 32.5)
	Scotland	38.1 (36.0, 40.3)	34.6 (32.1, 37.1)
	Wales	30.5 (27.8, 33.3)	33.4 (29.8, 37.2)
Prevalence of possible dependence, % (95% CI)	England	1.5 (1.2, 1.7)	1.3 (1.1, 1.5)
	Scotland	1.2 (0.8, 1.8)	1.3 (0.9, 1.9)
	Wales	1.6 (1.0, 2.5)	1.5 (0.7, 3.4)
Mean weekly alcohol consumption, units (95% CI)	England	6.2 (6.0, 6.4)	5.4 (5.2, 5.5)
	Scotland	6.4 (6.0, 6.8)	5.9 (5.6, 6.3)
	Wales	6.1 (5.6, 6.5)	5.8 (5.4, 6.3)

Table S8: Predicted time points and estimates when pre- and post-pandemic trends intersect. All estimates weighted and modelled assuming linear trends, among all adults ($N_{unweighted}$=208,010) and subgroups in England.

	Sample	Time point	Estimate
Prevalence of risky drinking	All	October 2034	22.8%
	Women	June 2032	16.3%
	Men	September 2036	29.0%
	Age 18-29	July 2033	29.5%
	Age 30+	March 2035	21.0%
	ABC1[1]	October 2025	32.1%
	C2DE[2]	*	*
Prevalence of possible dependence	All	November 2043	0.5%
Mean weekly alcohol consumption	All	June 2030	4.1 units
	Women	December 2027	2.9 units
	Men	October 2031	5.4 units
	Age 18-29	November 2041	2.3 units
	Age 30+	October 2029	4.4 units
	ABC1[1]	February 2026	5.3 units
	C2DE[2]	April 2056	0 units

[1] More advantaged social grades; [2] less advantaged social grades. * Trends will not intersect in the future.

6. Analysis adjusted for survey mode change

Based on a comparison of parallel-run face-to-face and telephone data collection,[1] the absolute difference in prevalence of risky drinking was -5.2 percentage points, in prevalence of possible alcohol dependence -0.5 percentage points, and in mean weekly alcohol consumption -0.7 units. When adding these adjustments to the telephone estimates, the step changes between February and April 2020 decreased to 10.5% (unadjusted: 30.3%), 34.1% (unadjusted: 90.2%), and 19.6% (unadjusted: 34.5%), respectively (Table S8). The step changes became even insignificant when applying the maximum mode effect based on the 95% CI of the absolute difference in estimates and approached the unadjusted step change when adjusting for the minimum mode effect. Table S9 provides sociodemographic characteristics of participants from the two surveys run in parallel in 2024.[1]

Table S9: Sociodemographic estimates from face-to-face (F2F) and telephone (Tel) data collected in 2022 and 2024 (N_{F2F}=4381 and N_{Tel}=4982).

Sociodemographic characteristics		Unweighted				Weighted			
		F2F, n	F2F, % (95% CI)	Tel, n	Tel, % (95% CI)	F2F, n	F2F, % (95% CI)	Tel, n	Tel, % (95% CI)
Age	16-24 years	602	13.7 (12.7-14.7)	507	10.2 (9.4-11.0)	574	13.1 (12.0-14.2)	657	13.2 (11.9-14.4)
	25-34 years	654	14.9 (13.8-16.0)	650	13.0 (12.1-13.9)	727	16.6 (15.4-17.8)	829	16.6 (15.3-18.0)
	35-44 years	687	15.7 (14.6-16.8)	630	12.6 (11.7-13.5)	687	15.7 (14.5-16.8)	778	15.6 (14.3-16.9)
	45-54 years	598	13.6 (12.6-14.6)	872	17.5 (16.4-18.6)	717	16.4 (15.1-17.6)	814	16.3 (15.2-17.5)
	55-64 years	699	16.0 (14.9-17.1)	855	17.2 (16.2-18.2)	675	15.4 (14.3-16.5)	762	15.3 (14.1-16.4)
	65+ years	1141	26.0 (24.7-27.3)	1465	29.4 (28.1-30.7)	1001	22.9 (21.6-24.1)	1138	22.9 (21.6-24.1)
	Refused	0	0 (0-0)	3	0.1 (0.0-0.2)	0	0 (0-0)	3	0.1 (0.0-0.1)
Gender	Men	2192	50.1 (48.6-51.6)	2493	50.3 (48.9-51.7)	2142	49.0 (47.4-50.5)	2417	48.8 (47.1-50.4)
	Women	2181	49.9 (48.4-51.4)	2440	49.2 (47.8-50.6)	2231	51.0 (49.5-52.6)	2516	50.7 (49.1-52.4)
	Non-binary	0	0 (0-0)	25	0.5 (0.3-0.7)	0	0 (0-0)	25	0.5 (0.3-0.7)
Social grade	AB	1014	23.1 (21.9-24.3)	1290	25.9 (24.7-27.1)	1155	26.4 (24.9-27.8)	1312	26.3 (24.9-27.8)
	C1	1586	36.2 (34.8-37.6)	2086	41.9 (40.5-43.3)	1298	29.6 (28.3-31.0)	1477	29.6 (28.3-31.0)
	C2	782	17.8 (16.7-18.9)	738	14.8 (13.8-15.8)	891	20.3 (19.0-21.6)	1010	20.3 (18.8-21.7)
	D	571	13.0 (12.0-14.0)	359	7.2 (6.5-7.9)	637	14.5 (13.4-15.7)	722	14.5 (13.0-16.0)
	E	428	9.8 (8.9-10.7)	509	10.2 (9.4-11.0)	400	9.1 (8.3-10.0)	460	9.2 (8.4-10.1)

Model specifications

- Family: gaussian
- Link function: identity
- Method: restricted maximum likelihood
- Optimizer: outer newton
- Smooth term: cyclic cubic regression spline
- Formula: log(outcome) ~ step + ramp + survey wave + s(seasonality, k = 12)

[1] Buss VH, Kock L, Tattan-Birch H, Jackson SE, Shahab L, Brown J. A comparison of prevalence estimates of smoking, alternative nicotine and alcohol use in Great Britain collected via telephone versus face-to-face: Smoking and Alcohol Toolkit surveys. *medRxiv* 2024: 2024.07.30.24311204.

Table S10: Parameter estimates for adjusted models using aggregated data.

	Step estimate (SE[1]), p-value	Ramp estimate (SE), p-value	Survey wave estimate (SE), p-value	s(seasonality) edf[2], p-value
Prevalence of risky drinking	0.100 (0.023), p<0.001	-0.002 (0.001), p=0.015	-0.001 (0.000), p=0.036	2.06, p=0.025
Prevalence of possible alcohol dependence	0.293 (0.121), p=0.017	-0.003 (0.003), p=0.447	-0.002 (0.002), p=0.298	2.12, p=0.014
Mean weekly alcohol consumption	0.179 (0.024), p<0.001	-0.003 (0.001), p<0.001	-0.001 (0.000), p<0.001	2.22, p=0.013

[1] Standard error; [2] estimated degrees of freedom.

Table S11: Weighted estimates for mode change adjustments of all three outcome measures.

	Prevalence of risky drinking	Prevalence of possible alcohol dependence	Mean weekly alcohol consumption
Face-to-face sample estimate (95% CI)[1]	25.3% (23.9, 26.7)	0.9% (0.6, 1.2)	4.7 (4.4, 5.1)
Telephone sample estimate (95% CI)[2]	30.4% (28.9, 32.0)	1.4% (1.0, 1.8)	5.5 (5.1, 5.9)
Absolute difference in estimates (95% CI)[3]	-5.2 (-7.0, -3.3)	-0.5 (-0.9, 0.0)	-0.7 (-1.2, -0.3)
Unadjusted step change (95% CI)[4]	30.3% (26.8, 33.8)	90.2% (62.2, 122.9)	34.5% (31.0, 38.0)
Adjusted step change (95% CI)[5]	10.5% (3.3, 16.4)	34.1 % (-24.2, 65.9)	19.6% (10.6, 24.5)

[1] Collected in March 2022 and between January and March 2024, sample size n=4332. [2] Collected in March 2022 and February 2024, sample size n= 4762. [3] Assessed using a two-sample t-tests comparing weighted data, with bootstrapped standard errors using 1000 bootstraps. [4] Step change from original model without adjusting for mode change.
[5] Generalised additive mode including monthly aggregated weighted estimate data, step function, ramp function, time variable, seasonality variable, and adjustment factor for mode change. Stimulation-based CI with adjustment factor simulated (n=1000) using mean and standard error for mode change adjustment factor.

Table S12: Predicted time and estimates when pre- and post-pandemic trends intersect, adjusted for mean mode effect.

	Time point	Estimate
Prevalence of risky drinking	February 2025	25.1%
Prevalence of possible dependence	December 2026	0.7%
Mean weekly alcohol consumption	September 2025	4.5 units

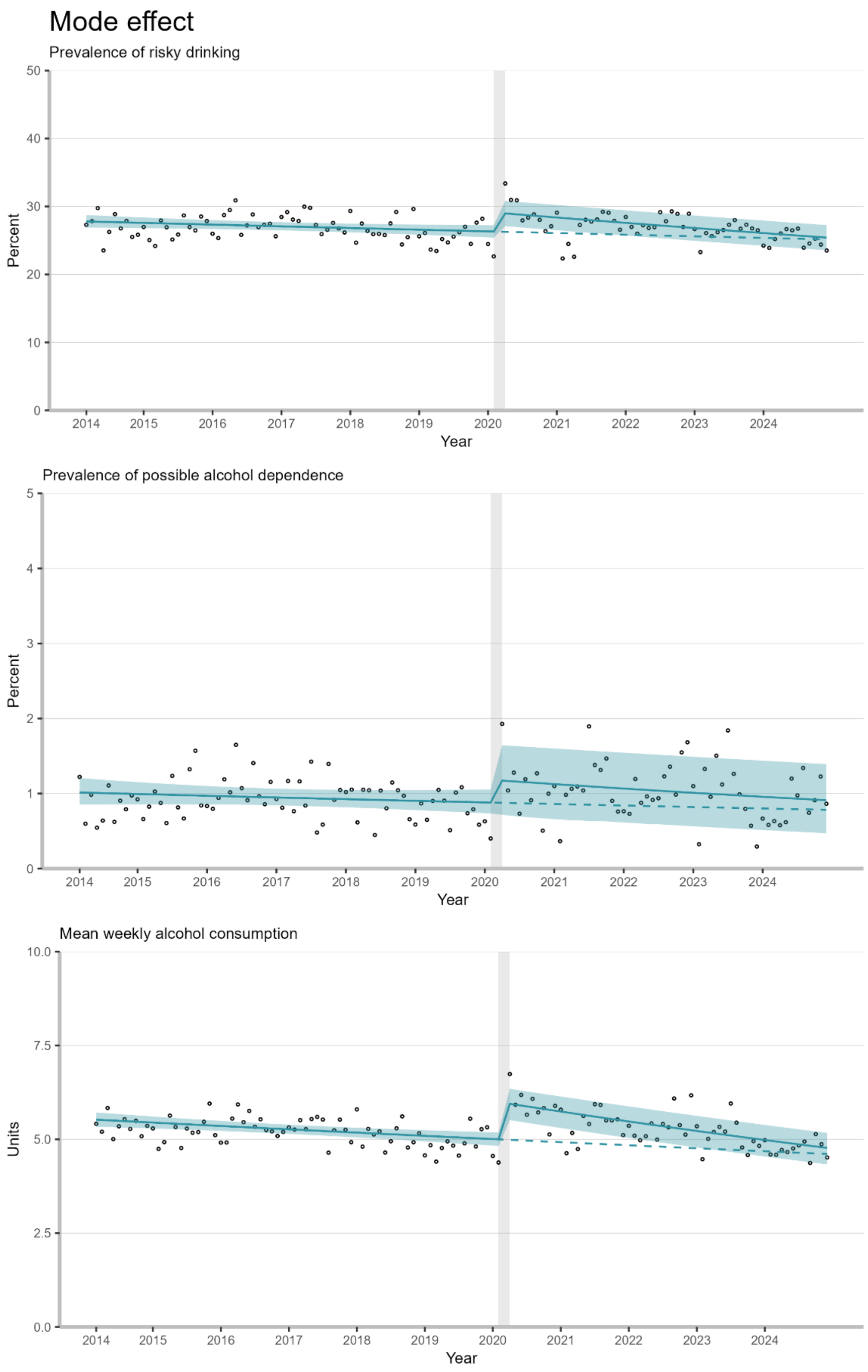

Figure S1: Trends in the adjusted weighted prevalence of risky drinking (top), adjusted weighted prevalence of possible alcohol dependence (middle), and adjusted weighted mean weekly alcohol consumption (bottom) among adults in England ($N_{unweighted}$=208,010) between 2014 and 2024 with a step change after February 2020 (indicated with vertical grey line between February and April 2020). Modelled using generalised additive models. Adjusted for mode change. Shaded areas indicate 95% CIs; created with adjustment factor simulated (n=1000) using mean and standard error for mode change adjustment factor. Dots show unmodelled values (three negative values for prevalence of possible alcohol dependence with max. mode effect not displayed) and dashed lines indicate continued pre-trends.

Table S13: Weighted estimates for mode change adjustments of all three outcome measures.

	**Subsample**	**Prevalence of risky drinking**	**Mean weekly alcohol consumption**
Absolute difference in estimates (95% CI)[3]	Women	-4.0 (-6.2, -1.7)	-0.6 (-1.0, -0.1)
	Men	-6.2 (-9.1, -3.3)	-0.6 (-1.4, 0.1)
	Aged 18-29	-3.0 (-7.6, 1.6)	-1.2 (-2.2, -0.2)
	Aged 30+	- 5.9 (-8.0, -3.9)	-0.6 (-1.1, -0.1)
	ABC1	-0.7 (-3.2, 1.7)	0.0 (-0.5, 0.6)
	C2DE	-10.8 (-13.7, -7.9)	-1.7 (-2.5, -1.0)
Unadjusted step change (95% CI)[4]	Women	40.5 (34.0, 47.4)	41.3 (36.1, 46.7)
	Men	25.0 (21.1, 29.0)	31.1 (26.5, 35.8)
	Aged 18-29	12.6 (6.6, 19.0)	25.6 (18.1, 33.6)
	Aged 30+	37.2 (33.0, 41.5)	35.6 (31.7, 39.6)
	ABC1	19.1 (15.5, 23.0)	25.3 (21.4, 29.4)
	C2DE	52.4 (44.9, 60.3)	51.2 (44.5, 58.2)
Adjusted step change (95% CI)[5]	Women	18.9 (6.3, 27.3)	24.7 (10.8, 32.2)
	Men	6.6 (-1.5, 14.0)	21.6 (9.8, 28.3)
	Aged 18-29	3.3 (-12.0, 16.7)	-2.0 (-25.9, 17.0)
	Aged 30+	12.8 (4.4, 19.8)	23.8 (13.3, 28.5)
	ABC1	16.7 (8.5, 22.0)	26.2 (16.1, 30.0)
	C2DE	-3.5 (-20.3, 10.8)	7.1 (-12.2, 22.7)

[1] Collected in March 2022 and between January and March 2024, sample size n=4332. [2] Collected in March 2022 and February 2024, sample size n= 4762. [3] Assessed using a two-sample t-tests comparing weighted data, with bootstrapped standard errors using 1000 bootstraps. [4] Step change from original model without adjusting for mode change.
[5] Generalised additive mode including monthly aggregated weighted estimate data, step function, ramp function, time variable, seasonality variable, and adjustment factor for mode change. Stimulation-based CI with adjustment factor simulated (n=1000) using mean and standard error for mode change adjustment factor.

Table S14: Predicted time and estimates when pre- and post-pandemic trends intersect, adjusted for mean mode effect.

		**Time point**	**Estimate**
Prevalence of risky drinking	Women	October 2025	17.3%
	Men	April 2025	32.6%
	Aged 18-29	August 2023	31.9%
	Aged 30+	February 2025	23.4%
	ABC1[1]	February 2025	32.1%
	C2DE[2]	March 2025	16.1%
Mean weekly alcohol consumption	Women	November 2024	3.1 units
	Men	September 2027	5.9 units
	Aged 18-29	*	*
	Aged 30+	May 2026	4.6 units
	ABC1[1]	March 2026	5.4 units
	C2DE[2]	August 2023	3.6 units

[1] More advantaged social grades; [2] Less advantaged social grades; * Trends will not intersect after start of pandemic.

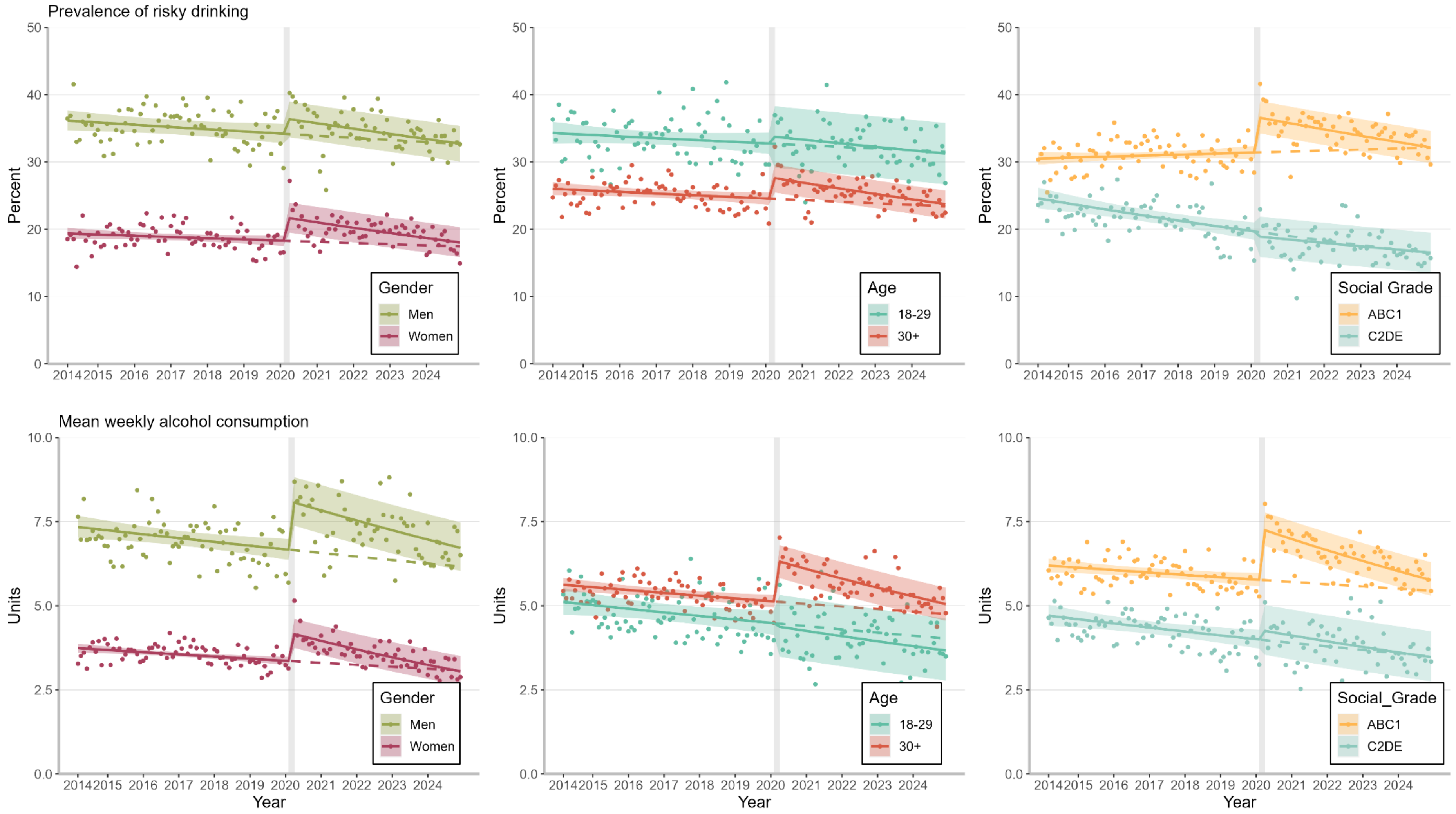

Figure S2: Trends in the adjusted weighted prevalence of risky drinking (top) and adjusted weighted mean weekly alcohol consumption (bottom) among adults in England ($N_{unweighted}$=208,010) between 2014 and 2024 with a step change after February 2020 (indicated with vertical grey line between February and April 2020) – stratified by gender (left), age (middle), and social grade (right). Modelled using generalised additive models. Adjusted for mode change. Shaded areas indicate 95% CIs; created with adjustment factor simulated (n=1000) using mean and standard error for mode change adjustment factor. Dots show unmodelled values (three negative values for prevalence of possible alcohol dependence with max. mode effect not displayed) and dashed lines indicate continued pre-trends. ABC1 indicated more advantaged social grades and C2DE less advantaged social grades.

7. Unweighted trends

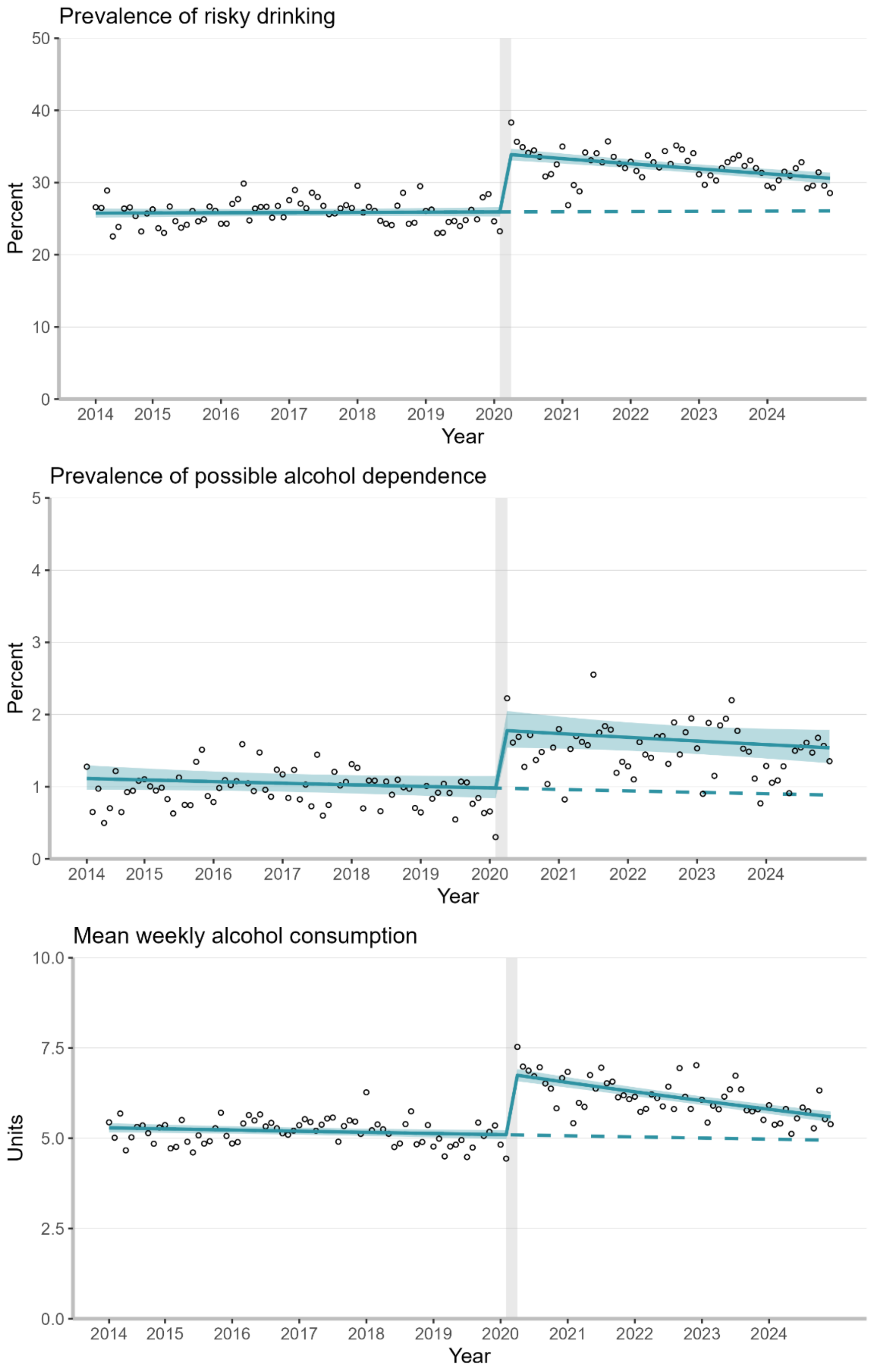

Figure S3: Trends in the unweighted prevalence of risky drinking (top), unweighted prevalence of possible alcohol dependence (middle), and unweighted mean weekly alcohol consumption (bottom) among adults in England ($N_{unweighted}$=208,010) between 2014 and 2024 with a step change after February 2020 (indicated with vertical grey line between February and April 2020). Modelled using generalised additive models. Shaded areas indicate 95% CIs. Dots show unmodelled values and dashed lines indicate continued pre-trends.

Table S15: Unweighted percentage changes in the prevalence of risky drinking and mean weekly alcohol consumption at the start of the COVID-19 pandemic among all in England ($N_{unweighted}$=208,010) and across subgroups.

	Pre vs. post, step change, % (95% CI)	Pre-COVID trend, %/year (95% CI)	Pre vs. post trend, Δ% (95% CI)
**Prevalence of risky drinking**			
All	30.8 (27.3, 34.4)	0.1 (-0.4, 0.7)	-2.3 (-3.1, -1.4)
Women	40.9 (34.1, 48.0)	-0.4 (-1.4, 0.6)	-1.9 (-3.8, 0.0)
Men	28.1 (24.1, 32.3)	0.5 (-0.1, 1.2)	-3.0 (-4.1, -2.0)
Age 18-29	10.4 (4.5, 16.7)	1.2 (0.2, 2.2)	-4.1 (-5.9, -2.3)
Age 30+	39.4 (35.2, 43.9)	-0.2 (-0.8, 0.5)	-1.9 (-2.9, -0.9)
ABC1[1]	22.3 (18.5, 26.2)	0.5 (-0.2, 1.2)	-3.3 (-4.3, -2.3)
C2DE[2]	53.3 (45.6, 61.5)	-3.1 (-4.0, -2.1)	1.6 (0.0, 3.4)
**Prevalence of possible dependence**			
All	81.8 (55.1, 113.2)	-2.1 (-5.4, 1.2)	-0.9 (-5.9, 4.2)
**Mean weekly alcohol consumption**			
All	32.9 (29.4, 36.4)	-0.6 (-1.1, -0.1)	-3.3 (-4.2, -2.5)
Women	44.9 (39.6, 50.5)	-0.8 (-1.6, -0.1)	-5.7 (-6.9, -4.5)
Men	31.0 (26.5, 35.8)	-0.3 (-1.0, 0.4)	-3.8 (-4.9, -2.6)
Age 18-29	21.0 (13.4, 29.1)	-0.7 (-1.9, 0.4)	-3.0 (-5.0, -0.9)
Age 30+	34.2 (30.4, 38.2)	-0.6 (-1.2, -0.1)	-3.2 (-4.2, -2.3)
ABC1[1]	25.8 (22.0, 29.6)	-0.9 (-1.5, -0.3)	-4.1 (-5.1, -3.2)
C2DE[2]	50.8 (43.3, 58.7)	-2.4 (-3.3, -1.5)	0.3 (-1.4, 2.0)

[1] More advantaged social grades; [2] less advantaged social grades.

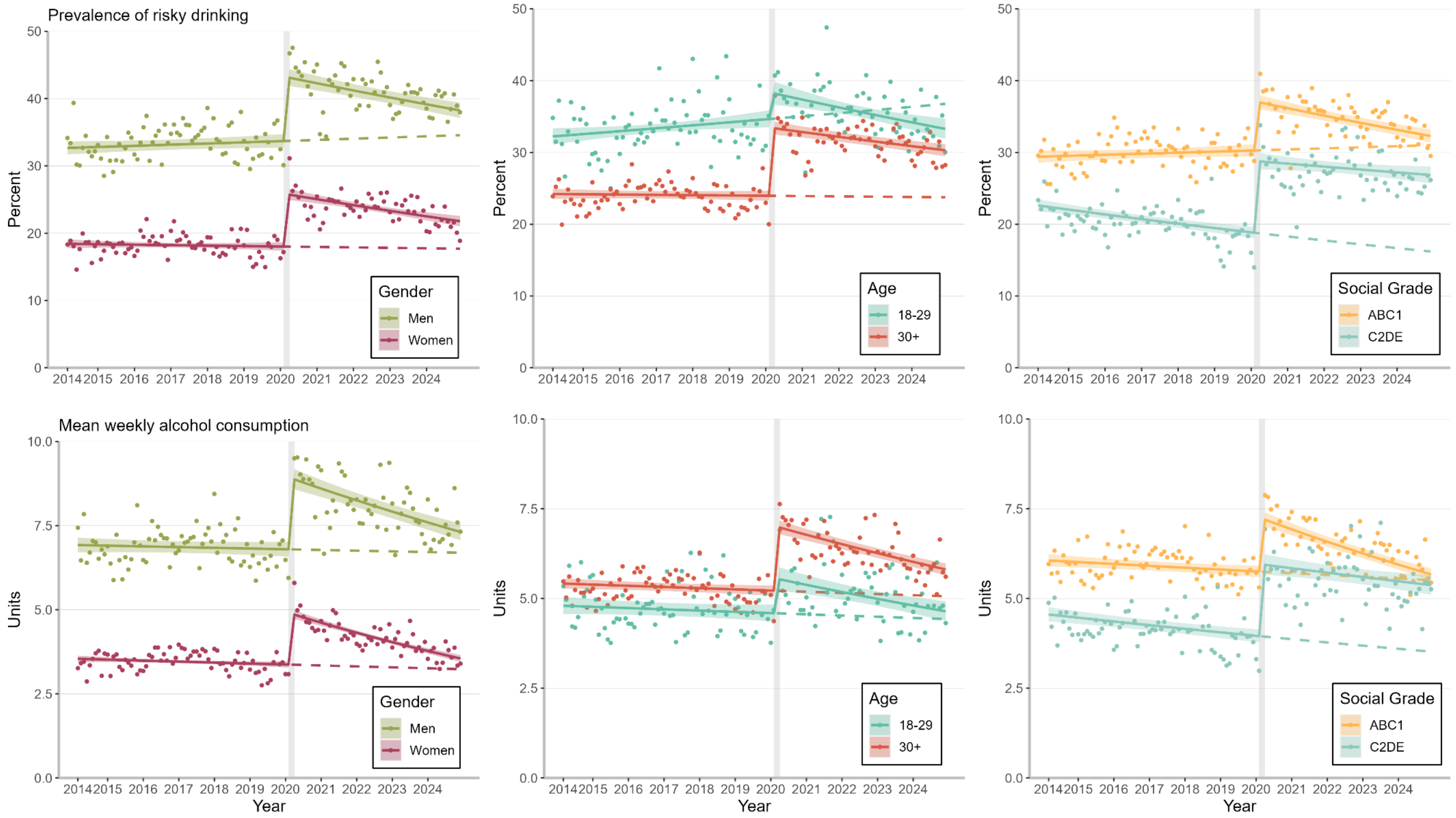

Figure S4: Trends in the unweighted prevalence of risky drinking (left) and unweighted mean weekly alcohol consumption (right) among adults in England ($N_{unweighted}$=208,010) between 2014 and 2024 with a step change after February 2020 (indicated with vertical grey line between February and April 2020) – stratified by gender (left), age (middle), and social grade (right). Modelled using generalised additive models. Shaded areas indicate 95% CIs. Dots show unmodelled values and dashed lines indicate continued pre-trends. ABC1 indicated more advantaged social grades and C2DE less advantaged social grades.

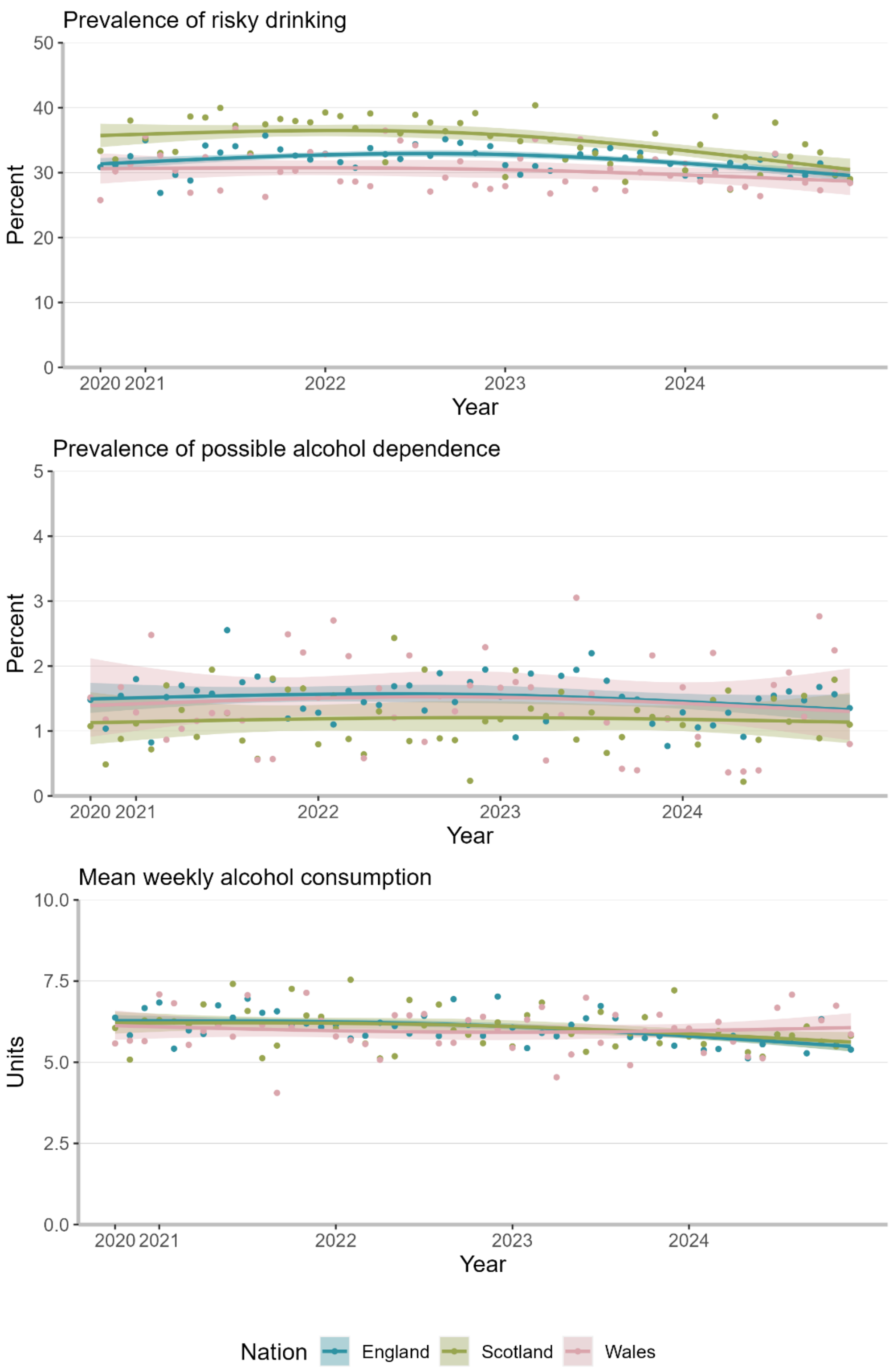

Figure S5: Trends in the unweighted prevalence of risky drinking (top), unweighted prevalence of possible alcohol dependence (middle), and unweighted mean weekly alcohol consumption (bottom) among adults England ($N_{unweighted}$=79,444), Scotland ($N_{unweighted}$=22,035), and Wales ($N_{unweighted}$=11,771) between 2020 and 2024. Modelled using restricted cubic splines with 3 knots. Shaded areas indicate 95% CIs. Dots show unmodelled values.

8. Mean weekly alcohol consumption adjusted for binge drinking

Mean weekly alcohol consumption adjusted for binge drinking, measured via AUDIT-C question 3 (see above). Binge drinking (having six or more standard drinks on one occasion) will be coded as:

- Never: 0,
- Less than monthly: 0.8019,
- Monthly: 2.75,
- Weekly: 11,
- Daily or almost daily: 33.

Weekly units of alcohol with adjustment will be calculated as drinking occasions per week times units consumed per drinking occasion plus binge drinking.

Table S16: Comparison of trends in the weighted mean weekly alcohol consumption without (left) and with adjusting for binge drinking (right) among adults in England ($N_{unweighted}$=208,010) between 2014 and 2024 with a step change after February 2020

	**Mean weekly alcohol consumption**	**Mean weekly alcohol consumption – adjusted for binge drinking**
Pre vs. post, step change, % (95% CI)	33.3 (29.6, 37.1)	34.7 (30.9, 38.6)
Pre-COVID trend, %/year (95% CI)	-1.6 (-2.1, -1.1)	-1.3 (-1.8, -0.7)
Pre vs. post trend, Δ% (95% CI)	-1.9 (-3.0, -0.8)	-1.7 (-2.8, -0.6)
Modelled estimate (in units) for March 2014	5.5 (5.3, 5.6)	7.0 (6.8, 7.2)
Modelled estimate (in units) for February 2020	5.0 (4.8, 5.1)	6.5 (6.3, 6.6)
Modelled estimate (in units) for April 2020	6.6 (6.4, 6.8)	8.7 (8.5, 8.9)
Modelled estimate (in units) for December 2024	5.8 (5.6, 5.9)	7.8 (7.5, 8.0)

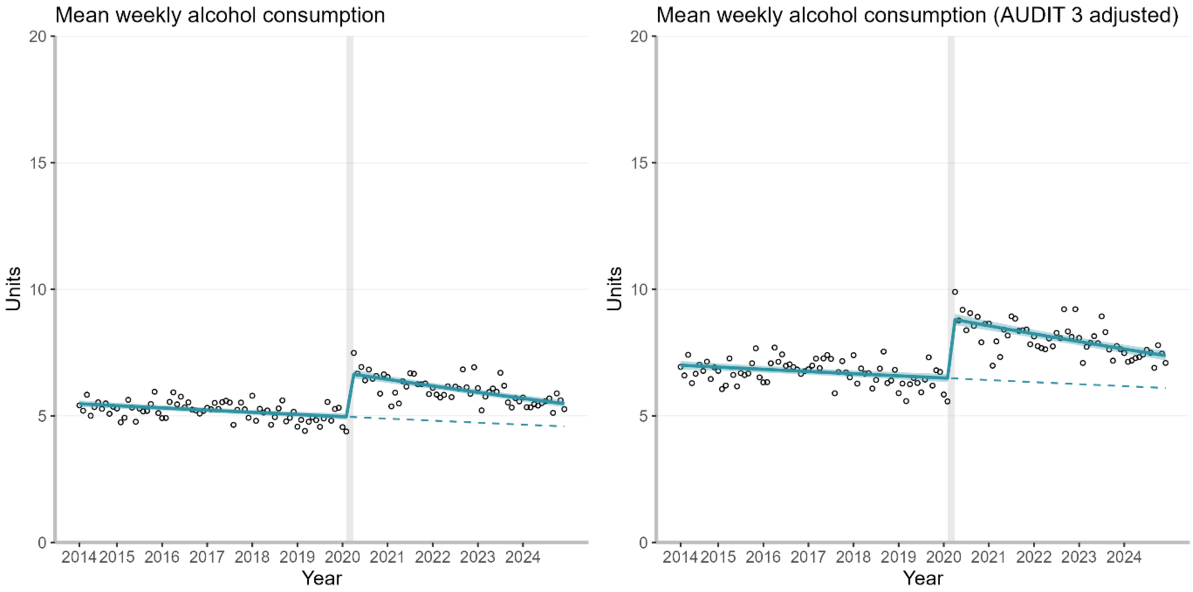

Figure S6: Comparison of trends in the weighted mean weekly alcohol consumption without (left) and with adjusting for binge drinking (right) among adults in England ($N_{unweighted}$=208,010) between 2014 and 2024 with a step change after February 2020 (indicated with vertical grey line between February and April 2020). Modelled using generalised additive models. Shaded areas indicate 95% CIs. Dots show unmodelled values and dashed lines indicate continued pre-trends.
